# Bell's Palsy as a Late Neurologic Manifestation of COVID-19 Infection

**DOI:** 10.7759/cureus.13881

**Published:** 2021-03-14

**Authors:** Ibiyemi O Oke, Olubunmi O Oladunjoye, Adeolu O Oladunjoye, Anish Paudel, Ryan Zimmerman

**Affiliations:** 1 Internal Medicine, Reading Hospital - Tower Health, West Reading, USA; 2 Medical Critical Care, Boston Children's Hospital, Boston, USA; 3 Psychiatry, Reading Hospital - Tower Health, West Reading, USA; 4 Internal Medicine, Reading Hospital - Tower health, West Reading, USA

**Keywords:** bell’s palsy, covid-19 infection, facial weakness, neurologic manifestation

## Abstract

Bell’s palsy is acute peripheral facial nerve palsy; its cause is often unknown but it can be triggered by acute viral infection. Coronavirus disease 2019 (COVID-19) infection commonly presents with respiratory symptoms, but neurologic complications have been reported. A few studies have reported the occurrence of facial nerve palsy during the COVID-19 pandemic. We present a case of Bell's palsy in a 36-year-old man with COVID-19 infection and a past medical history of nephrolithiasis. He presented to the emergency room with a day history of sudden right facial weakness and difficulty closing his right eye four weeks following a diagnosis of COVID-19 infection. Physical examination revealed right lower motor neuron facial nerve palsy (House-Brackmann grade IV). Serologic screen for Lyme disease, human immunodeficiency virus (HIV), and herpes simplex virus (HSV) 1 and 2 were negative for acute infection; however, neuroimaging with MRI confirmed Bell's palsy. He made remarkable improvement following treatment with a course of valacyclovir and methylprednisolone. This case adds to the growing body of literature on neurological complications that should be considered when managing patients with COVID-19 infection.

## Introduction

Bell’s palsy is an acute peripheral lower motor neuron (LMN) facial nerve palsy leading to weakness on one side of the face without any other neurologic abnormalities on examination. The cause is often unknown; however, herpes simplex virus isoform 1 (HSV 1) and/or herpes zoster virus (HZV) reactivation is thought to be the most likely cause [1]. Severe acute respiratory syndrome coronavirus 2 (SARS-CoV-2) is the novel virus that causes coronavirus disease 2019 (COVID-19). It was first identified in Wuhan, a city in Hubei province of China, in December 2019.

There are a few theories on the neuropathogenesis of COVID-19, which include the binding of coronavirus to angiotensin-converting enzyme 2 (ACE2) receptors, which are widely distributed on glial cells and neurons [2,3]. Dubé et al. postulated in their study with animal models that there is axonal transport of human coronavirus (HCoV) OC43 protein into the nervous system [4]. These two mechanisms may lead to nerve damage through direct injury, autoimmunity, and ischemia of the vasa nervorum or inflammatory demyelination [5,6].

Facial nerve palsy may be the first presentation of COVID-19 and it may occur within a few days of its diagnosis [7-13]. We present a patient with a unilateral LMN facial nerve palsy four weeks after a diagnosis of COVID-19 infection.

## Case presentation

**Figure 1:**
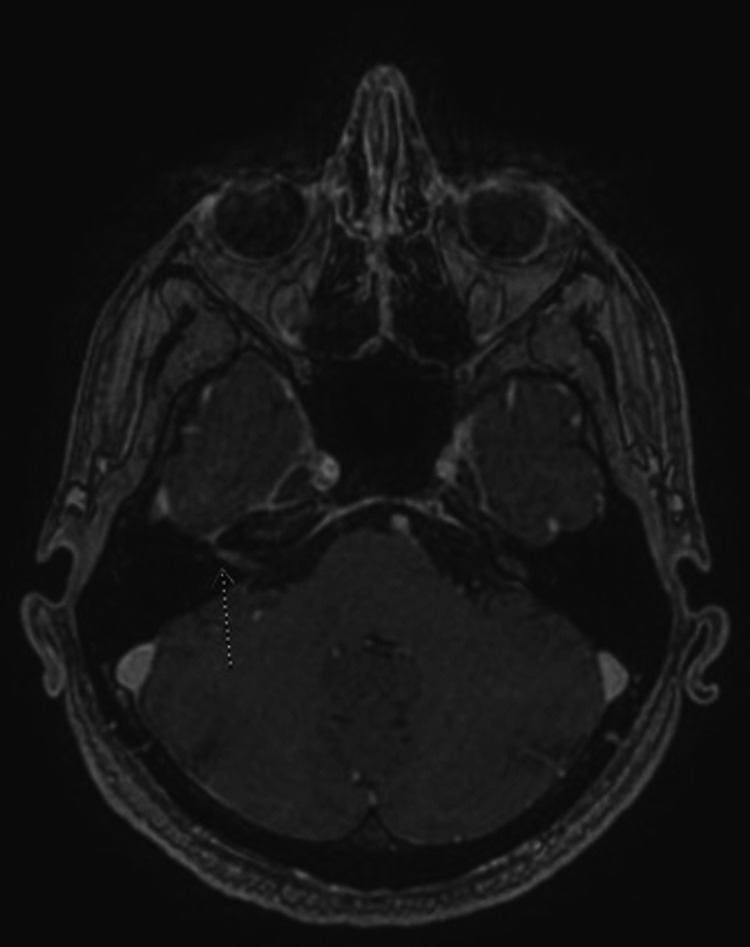
Magnetic resonance imaging (MRI) of the brain Arrow shows asymmetric enhancement of the right facial nerve

A 36-year-old man with a past medical history of nephrolithiasis presented to the emergency room with a day history of sudden right facial weakness and difficulty closing his right eye four weeks following a diagnosis of COVID-19 infection. He had no other neurologic symptoms and no reports of earache, rash, flu-like symptoms, or recent cold sore. He had fever and body aches when he was tested for COVID- 19, which resolved after a few days of treatment with acetaminophen. He denied cough, shortness of breath, or fatigue, and his last chest radiograph was unremarkable. His vital signs on admission were as follows: heart rate of 75 beats/min, blood pressure of 134/97 mmHg, respiratory rate of 11 breaths/min, and temperature of 36.8oC. Physical examination revealed right LMN facial nerve palsy (House-Brackmann grade IV), but no vesicles or scabs were seen around the external ear and he had no oral ulcers. Laboratory tests showed normal complete blood count and basic metabolic panel, non-reactive human immunodeficiency virus (HIV) 1 and 2, negative herpes simplex virus (HSV) 2 immunoglobulin G (IgG), positive HSV 1 IgG, negative HSV immunoglobulin M (IgM), and Lyme IgG/IgM antibody. He was not retested for COVID-19. Computed tomography (CT) of the brain was negative for stroke or any other intracranial abnormality. Magnetic resonance imaging (MRI) brain showed asymmetric enhancement of the right facial nerve consistent with Bell’s palsy (Figure 1).

A diagnosis of Bell’s palsy was made, and he was discharged on a seven-day course of oral valacyclovir 1,000 mg twice daily and a tapering dose of methylprednisolone (24 mg on day 1, 20 mg on day 2, 16 mg on day 3, 12 mg on day 4, 8 mg on day 5, and 4 mg on day 6). He was provided with eye patch and artificial tears. At three-month follow-up, he reported significant improvement in symptoms and he had only mild right facial nerve weakness (House-Brackmann grade II) on examination.

## Discussion

COVID-19 infection presents mostly with fever and pulmonary symptoms, which could range from mild cough to acute respiratory distress syndrome (ARDS) [14]. Notable complications and causes of death in these patients include sepsis, acute kidney injury, ARDS, acute hypoxic encephalopathy, and acute cardiac injury [14].

Increasing number of COVID-19 related facial nerve palsies are now being reported, with most being the first presenting symptom or occurring within the first week of onset of viral symptoms or a positive COVID-19 test [7-13]. This is a rare case of facial nerve palsy presenting three weeks after resolution of viral symptoms and four weeks after a positive COVID-19 reverse-transcription polymerase chain reaction (RT-PCR) test.

Over the past year, we have come to know that COVID-19 may also present with neurologic complications such as anosmia, dysgeusia, encephalopathy, Guillain-Barre syndrome, Miller-Fisher syndrome, and polyneuritis cranialis [14-17]. Like this 36-year-old patient, Bell’s palsy and CoV is predominantly seen in adults and is often unilateral. Theophanous et al. reported a case in a six-year-old child [9] and Khaja et al. documented a rare case of COVID- 19 and bilateral facial palsy [18]. Although this patient had a positive HSV1 IgG antibody, his IgM antibody was negative and he had no HSV viral exanthem. It still remains unknown if there is a relationship between COVID-19 infection and reactivation of HSV infection leading to Bell’s palsy.

The standard treatment of Bell’s palsy is steroid with or without antiviral, and this has proven to be effective [19]. A large review by Peitersen et al. shows that 85% of patients’ function was returned within three weeks and the remaining 15% after three to five months [20]. The exact reason for the delayed return to function in the latter group is still not clear, but there is a suggestion that the presence of facial nerve enhancement on MRI may be associated with a longer duration of symptom. Our patient had positive MRI enhancement and received a course of steroid and valacyclovir, and at his three-month follow-up visit, he had only mild facial weakness.

## Conclusions

Facial nerve palsy can be seen any time during the course of COVID-19 infection and can sometimes present as late as one month after diagnosis. So far, we believe its association with COVID-19 is more than just a mere coincidence but one of the emerging manifestations of this novel virus. Clinicians should consider this as a potential neurologic complication when managing patients with COVID-19 infection and start treatment early when present, as they would in other causes of Bell's palsy.
